# *Capnocytophaga canimorsus* peritonitis diagnosed by mass spectrometry in a diabetic patient undergoing peritoneal dialysis: a case report

**DOI:** 10.1186/s12882-019-1415-x

**Published:** 2019-06-14

**Authors:** Katsuyuki Tanabe, Shugo Okamoto, Sumie Hiramatsu Asano, Jun Wada

**Affiliations:** Department of Nephrology, Rheumatology, Endocrinology and Metabolism, Okayama University Graduate School of Medicine, Dentistry and Pharmaceutical Sciences, 2-5-1 Shikata-cho, Kita-ku, Okayama, 700-8558 Japan

**Keywords:** Peritoneal dialysis, Peritonitis, *Capnocytophaga canimorsus*, MALDI-TOF mass spectrometry

## Abstract

**Background:**

Bacterial peritonitis is a serious complication of patients undergoing peritoneal dialysis (PD). Although the identification of causative organisms and use of appropriate antibiotics are essential for treatment, rare and fastidious bacteria are sometimes difficult to detect by conventional biochemical assays. *Capnocytophaga canimorsus* is a fastidious and slow-growing bacterium that forms a part of the normal oral flora of dogs and cats and is extremely rare as a peritonitis-causing organism. This report demonstrates the usefulness of a mass spectrometry-based technique in identifying such a rare organism in PD-related peritonitis and discusses the diagnosis and treatment of *C. canimorsus* peritonitis.

**Case presentation:**

A 49-year-old woman with type 2 diabetes mellitus underwent PD for two years. Repeated exit-site infections led to subcutaneous pathway diversion two months ago. She was hospitalized with fever and abdominal pain as well as cloudy dialysis effluent. Laboratory data revealed increased serum C-reactive protein level and white blood cell (WBC) count in the effluent. Her exit site had no sign of infection, leading to the diagnosis of PD-related peritonitis. Initial therapy with intraperitoneal ceftazidime immediately ameliorated her symptoms, and the WBC count in the effluent normalized in five days. Culture test results of the dialysis effluent on admission were negative with no information regarding the infection route. However, mass spectrometry (MALDI Biotyper, Bruker Daltonics) successfully obtained the specific spectral pattern for *C. canimorsus*. She had four cats in her house and was advised not to allow the cats in the room where the bag exchange took place.

**Conclusions:**

*C. canimorsus* is a rare cause of peritonitis in PD patients and is usually susceptible to intraperitoneal third-generation cephalosporins. This mass spectrometry-based bacterial identification method could provide more opportunities to identify uncommon causes and promote appropriate antibiotics therapy in PD-related peritonitis.

## Background

Bacterial peritonitis is a serious complication in end-stage renal disease patients undergoing peritoneal dialysis (PD). Bacterial peritonitis is associated with an increased risk of PD withdrawal [1]. The pathogenesis of PD-related peritonitis depends on the type of causative pathogens. Among various organisms, *Staphylococcus aureus* and *S. epidermis* are the major peritonitis-causing bacteria in PD patients; peritonitis caused by these organisms is generally associated with the exit site of a peritoneal catheter and subcutaneous tunnel infections. Conversely, the source of gram-negative bacteria in PD-related peritonitis is often presumed to be the gastrointestinal tract. However, the identification of causative organisms is sometimes challenging [2], especially if the causes are rare or fastidious organisms. Because negative dialysate culture in patients with peritonitis would limit mapping out the preventive strategies against the recurrence, the development of innovative bacterial identification techniques is warranted.

*Capnocytophaga canimorsus* is a gram-negative bacillus that is a part of the normal oral flora of dogs and cats [3]. Therefore, it could be the potential causative organism in pet-induced peritonitis in PD patients who have cats or dogs in their house. *Pasteurella multocida*, another representative component of the oral flora of many animals, is a peritonitis-causing organism in PD patients as reported in many case reports and series [4]. Nevertheless, PD-related *C. canimorsus* peritonitis is extremely rare, because it is usually difficult to identify the organism using traditional biochemical assays [5], leading to the diagnosis of culture-negative peritonitis. In this report, we present a case of *C. canimorsus* peritonitis in a woman with diabetes who was undergoing PD and was diagnosed using a mass spectrometry-based technique; we also discuss the utility of this novel bacterial identification method in PD-related peritonitis as well as the diagnosis and treatment of *C. canimorsus* peritonitis.

## Case presentation

**Fig. 1:**
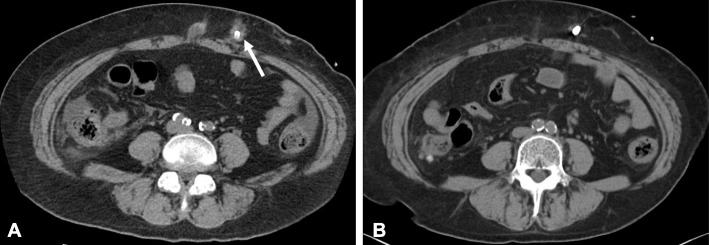
Abdominal computed tomography images in this patient. Images were obtained before subcutaneous pathway diversion (**a**) and at admission for peritonitis (**b**). Subcutaneous abscess around the catheter is indicated by arrow in **a**

A 49-year-old woman was admitted because of fever and abdominal pain. She had chronic renal failure caused by type 2 diabetes mellitus and initiated continuous ambulatory PD (CAPD) one year ago, with a conventional twin-bag system and no automated cycler device. Although her body fluids and solute levels were well controlled, she developed recurrent infections with *Staphylococcus caprae* at the catheter exit site, leading to chronic subcutaneous tunnel infection with abscess around the catheter (Fig. 1a). Subsequently, she underwent subcutaneous pathway diversion two months ago. When admitted to the hospital, she had a body temperature of 38.6 °C, pulse rate of 98 beats/minute, and blood pressure of 118/73 mmHg. Her entire abdomen was tender, with apparent rebound tenderness. The catheter exit site showed no signs of infection. Her laboratory data revealed that a white blood cell (WBC) count of 8950/μL, with 85.1% neutrophils, and C-reactive protein level of 9.43 mg/dL. Her dialysis effluent appeared cloudy, and the WBC count in the effluent was 3870/μL (76% polymorphonuclear cells). Considering these results, she was diagnosed as having CAPD-associated peritonitis.

**Fig. 2:**
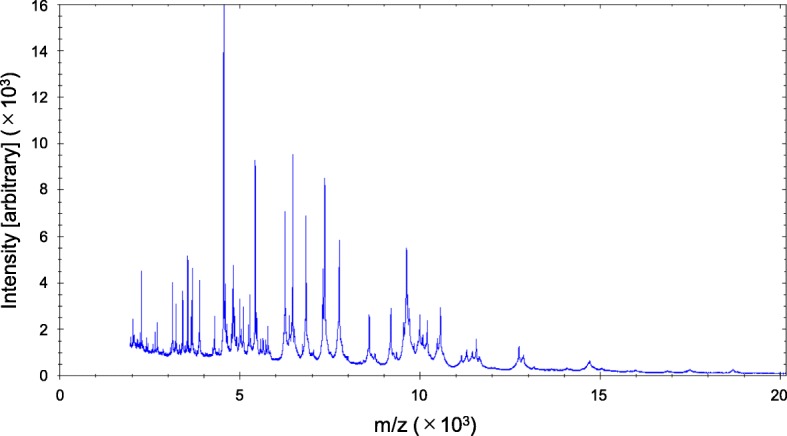
Mass spectral image obtained from a colony, indicating specific spectral pattern for *Capnocytophaga canimorsus*

Initially, the peritonitis was suspected to be because of the recurrent subcutaneous tunnel infection caused by *S. caprae*, as it occurred relatively soon after the subcutaneous pathway diversion. However, an abdominal computed tomography scan revealed no findings of recurrent subcutaneous abscess (Fig. 1b). After sampling the effluent in blood culture bottles and sterile plastic tubes for bacterial culture, she received empiric antibiotic therapy with continuous intraperitoneal ceftazidime that was mixed in the dialysate bags (125 mg/L of dialysate). Her fever and abdominal pain immediately ameliorated, and the WBC count in the effluent normalized in five days. The causative bacteria for the peritonitis were not identified by culture testing. However, mass spectrometry (MALDI Biotyper, Bruker Daltonics, Germany) for bacterial identification successfully detected *C. canimorsus* in a tiny colony in solid culture medium inoculated from samples collected in sterile plastic tubes (Fig. 2). The patient had four cats in her house, and the room for bag exchange was not sequestered from these cats. Additionally, she frequently slept with the cats on the bed, although the catheter had never been damaged by cat bite or scratch. She was thoroughly advised not to allow the cats in her bedroom and in the room for bag exchange.

## Discussion and conclusions

Bacterial peritonitis is a serious complication of PD. It is generally diagnosed by characteristic symptoms such as fever and abdominal pain as well as cloudy dialysis effluent. Because catheter exit site and subcutaneous tunnel infections are the major contributing factors to PD-related peritonitis, exit site care is important as a preventive strategy against peritonitis [6]; moreover, chronic tunnel infection frequently needs subcutaneous pathway diversion or catheter removal [7]. In the present case, peritonitis was initially suspected to be caused by a persisting tunnel infection. Therefore, if *S. caprae* was detected by culture testing in the effluent, she would have been diagnosed with PD-related peritonitis induced by refractory tunnel infection after the diversion, and catheter removal would have been considered. However, the culture test results were negative and did not provide information regarding the route of infection. Appropriate antibiotic therapy for peritonitis and preventive strategies against recurrence depend on the identification of causative organisms by culture test [8]. However, the identification is sometimes challenging. Using blood culture bottles recommended by the International Society for Peritoneal Dialysis (ISPD) guideline, culture negative peritonitis is found in 10–20% of cases [8]. Therefore, various innovative techniques such as 16S rRNA sequencing and mass spectrometry have been introduced in clinical practice to improve the identification of rate causative organisms.

Mass spectrometry is an analytical technique that determines the mass-to-charge ratio (m/z) of ions to identify and quantitate molecules. The development of matrix-assisted laser desorption ionization-time of flight mass spectrometry (MALDI-TOF-MS) has expanded the applicability to various fields [9]. MALDI can ionize molecules in samples to make them positively charged, and the charged molecules are separated according to their own TOF. This technique has emerged as a novel tool for identifying bacterial genus and species in clinical practice [9]. Using a single colony of organism, MALDI-TOF-MS can generate a bacterial species-specific spectrum called peptide mass fingerprint (PMF), as shown in Fig. 2, and PMF is automatically matched to an existing database to determine the species. This system enables fast and accurate bacterial identification based on only a fraction of a single colony. In addition, this technique has better cost-effective performance compared with 16S rRNA sequencing [10]. Although very low running cost has enabled the routine use of this system in many academic hospitals and tertiary medical centers, the high cost of the equipment has limited its introduction in most ordinary hospitals, especially in developing countries. Some researchers have introduced MALDI-TOF-MS to detect rare peritonitis-causing bacteria in patients undergoing PD. Lam et al. reported four cases of PD-related peritonitis caused by *Gordonia* species, an extremely rare peritonitis-causing pathogen, using both 16S rRNA sequencing and MALDI-TOF-MS [11]. In addition, another group successfully identified *Arthrobacter sanguinis* in the effluent from a PD patient with peritonitis, which has been the only reported case of peritonitis caused by this organism [12]. Thus, MALDI-TOF-MS may be a powerful method to resolve the diagnostic challenge owing to the difficulty in bacterial identification by conventional culture and biochemical assays for PD-related peritonitis. However, there is insufficient evidence to support the routine use of MALDI-TOF-MS in PD patients, as stated in the ISPD guideline [8]. We have routinely used MALDI-TOF-MS in parallel with the conventional culture test in patients with infectious diseases. However, whenever culture tests provide positive results, they are prioritized over MALDI-TOF-MS. Only when the culture test fails to detect pathogens, MALDI-TOF-MS is used in the diagnosis and treatment of infectious diseases. Therefore, this technique should be considered as a compensatory tool to the conventional culture test until the superiority of MALDI-TOF-MS is demonstrated in future studies.

Among *Capnocytophaga* species, *C. canimorsus* and *C. cynodegmi* are well-known components of the normal flora of dogs and cats [3]. In humans, these bacteria generally cause localized skin infections in patients who received dog bites or cat scratches. However, *C. canimorsus* causes sepsis, especially in patients with prior splenectomy, or meningitis [13]. A recent report described severe sepsis with thrombotic microangiopathy caused by *C. canimorsus* in an immunocompetent patient [14]. *Capnocytophaga* species are extremely rare as the causative organism of PD-related peritonitis. Based on a PubMed search, there have been only six cases of peritonitis caused by *Capnocytophaga* species in PD patients (Table 1). However, given that pet-related peritonitis, especially *P. multocida* peritonitis, is not uncommon, *Capnocytophaga* species-induced peritonitis may actually occur more often. Its characteristics of fastidiousness and slow growth may result in negative culture test results in most cases. In the previous three reports, conventional culture and biochemical assays could identify *Capnocytophaga* species as peritonitis-causing pathogen in PD patients [5, 15, 16], only if enough numbers of bacteria grew up on the culture medium. However, the species could not be determined in all three cases. Thus far, only two cases of *C. canimorsus*-induced peritonitis in PD patients have been reported in 1999 and 2013 [17, 18]. Unfortunately, the identification methods were not described in the former case report. The latter case identified *C. canimorsus* using 16S rRNA sequencing. Another report also successfully identified *C. cynodegmi* in PD patients with peritonitis using 16S rRNA sequencing [19]. Therefore, this report describes the first case that was diagnosed as *C. canimorsus*-induced peritonitis by mass spectrometry. Given the limited utility of 16S rRNA sequencing in hospitals, the application of MALDI-TOF-MS in clinical laboratories may augment opportunities for detecting *C. canimorsus* in PD-related peritonitis.

**Table 1:** Summary of *Capnocytophaga* species peritonitis in PD patients

	Patient	PD duration	Species	Identification	Treatment	Outcome
Esteban et al. (1995) [5]	73 y, M	3 years	*C. sputigena* (putative)	Conventional biochemical assay with other laboratory tests	VCM + GM → ABPC + CPFX	Improved
Chadha et al. (1999) [17]	18 y, M	5 years	*C. canimorsus*	Unspecified	IP CEZ + TOB → IP TIPC + IV TOB + VCM → IP TIPC + CLDM	Catheter removal
Pers et al. (2007) [19]	67 y, M	2 years	*C. cynodegmi*	16S rRNA gene sequencing	IP GM + VCM → IV CXM + GM + MDZ	Catheter removal
Chow et al. (2010) [15]	39 y, F	3 years	Unknown	Unspecified	IP CEZ + CAZ	Improved
Sadjadi et al. (2012) [16]^a^	83 y, M	2 years	Unknown	Conventional biochemical assay	IP VCM + CAZ (+ oral CPFX)	Catheter removal
Al-Fifi et al. (2013) [18]^a^	49 y, M	1 year	*C. canimorsus*	16S rRNA gene sequencing	IP CEZ + TOB → IP CEZ + CPFX	Improved
Present case	49 y, F	1 year	*C. canimorsus*	MALDI-TOF mass spectrometry	IP CAZ	Improved

*IP* intraperitoneal, *IV* intravenous, *VCM* vancomycin, *GM* gentamycin, *ABPC* ampicillin, *CPFX* ciprofloxacin, *CEZ* cephazolin, *TOB* tobramycin, *TIPC* ticarcillin, *CLDM* clindamycin, *CXM* cefuroxime, *MDZ* metronidazole, *CAZ* ceftazidime, *MALDI-TOF* matrix-assisted laser desorption ionization-time of flight

^a^These cases were co-infected with *Pasteurella multocida*

Even though MALDI-TOF-MS may facilitate the detection of *C. canimorsus*, preventive strategies are more important than diagnosis and treatment in domestic animals or pet-associated peritonitis. The ISPD guideline states that domestic animals should be excluded from the space where PD is being performed [8]. We have also advised all PD patients, including our present patient, not to allow their pets in the room for bag exchange. However, a previous study of *Pasteurella* species-induced peritonitis showed that 10 of 37 patients had no contact between their pets and the PD treatment area [20], suggesting that the preventive strategy was inadequate to avoid the development of peritonitis. As diagnosis of pet-associated peritonitis can lead to re-education of PD patients, bacterial identification should be important for secondary prevention.

Antibiotics susceptibility testing of *Capnocytophaga* species is difficult because of its fastidious nature. Novel bacterial identification techniques, including 16S rRNA sequencing and MALDI-TOF-MS, intrinsically provide no information of antimicrobial susceptibility. However, common knowledge of susceptibility of *Capnocytophaga* species to antibiotics is available in clinical practice. Based on previous reports, *C. canimorsus* is usually sensitive to various antibiotics such as penicillin, ampicillin, third-generation cephalosporins (for example, cefotaxime), clindamycin, erythromycin, and ciprofloxacin, but is resistant to aminoglycoside and trimethoprim-sulfamethoxazole [21]. Although *C. canimorsus* is susceptible to various classes of antibiotics, the outcome of peritonitis caused by the organism is not always desirable, as listed in Table 1. Most common empiric therapy for PD-related peritonitis consists of cefazolin or vancomycin and aminoglycosides to cover gram-positive and Gram-negative bacteria, respectively [22]. However, such combinations of antibiotics have often failed to improve the peritonitis caused by *Capnocytophaga* species and have led to transient or permanent withdrawal from PD (Table 1). As in the present case and another report [15], intraperitoneal ceftazidime appears to be a preferable first-line therapy for *C. canimorsus* peritonitis, although antibiotic therapy, including ceftazidime, resulted in the incomplete resolution of peritonitis and subsequent PD withdrawal in one case co-infected with *P. multocida* and *Enterobacter cloacae* [18]. Overall, early conversion from aminoglycoside-based empiric therapy to third-generation cephalosporins or ciprofloxacin may be an efficient treatment strategy for *C. canimorsus* peritonitis. Further accumulation of case reports would be necessary to confirm the efficacy of the treatment strategy. Considering that MALDI-TOF-MS may enable faster identification of fastidious and slow-growing organisms in the conventional culture test, this technique may initiate early therapeutic strategies in other cumbersome cases of peritonitis caused by pathogens that need much longer time and special media for the culture test. A previous study successfully identified *Candida* species and *Mycobacterium tuberculosis* as pathogens in PD-related peritonitis using MALDI-TOF-MS [23]. Therefore, faster identification by MALDI-TOF-MS may enable the introduction of appropriate therapeutic strategies in the earlier stage and improve patient prognosis in peritonitis.

In conclusion, *Capnocytophaga* species should be considered as peritonitis-causing organisms in PD patients, especially those who have dogs or cats in their house. Novel techniques such as mass spectrometry would provide more opportunities to identify uncommon causes in PD-related peritonitis, as well as establish preventive strategies against the recurrence.

## Data Availability

All data analyzed during this report are included in this published article.

## Abbreviations

CAPD: Continuous ambulatory peritoneal dialysis
ISPD: International Society for Peritoneal Dialysis
MALDI-TOF-MS: Matrix-assisted laser desorption ionization-time of flight mass spectrometry
PD: Peritoneal dialysis
PMF: Peptide mass fingerprint
WBC: White blood cell
